# Exploring Multigenerational Relationships and Episodic Memory in Later Life: A Study of Childhood Family Dynamics

**DOI:** 10.1093/geroni/igaf122.2561

**Published:** 2025-12-31

**Authors:** Kimson Johnson, Maddison Linker

**Affiliations:** University of Michigan Institute for Social Research, Ann Arbor, Michigan, United States; University of Michigan, Ann Arbor, Michigan, United States

## Abstract

**Introduction:**

Understanding how childhood family dynamics shape cognitive health in later life is important for addressing cognitive decline, yet this topic remains understudied. This study investigates the impact of parental relationship quality and grandparental co-residence on episodic memory in older adults, exploring whether living with grandparents in childhood buffers the negative cognitive effects of poor parental relationships.

**Methods:**

Data from the Health and Retirement Study (HRS) 2015-2017 Life History Mail Survey, the Childhood Family and Health, and the 2020 Cross-Wave Imputation of Cognitive Functioning Measures were used (n = 3,991; Mage = 71.9, SE = 0.27). Parental relationship quality and grandparental co-residence were categorized into four groups: (1) good parental relationship, lived with grandparents; (2) good parental relationship, did not live with grandparents; (3) poor parental relationship, lived with grandparents; (4) poor parental relationship, did not live with grandparents. Episodic memory was measured using total recall (range: 0–20). Linear regression models adjusted for sociodemographic factors, childhood health, childhood SES, household size, and survey weights.

**Results:**

Compared to those with good parental relationships and grandparent co-residence, participants with good parental relationships and without grandparental co-residence had lower episodic memory scores (β = -0.52, SE = 0.18, p < .01). Participants with poor parental relationships and grandparent co-residence had even lower scores (β = -1.02, SE = 0.47, p < .05).

**Conclusion:**

Findings suggest that poor parental relationships negatively impacts episodic memory, even with grandparental co-residence, emphasizing the importance of considering both parental dynamics and multigenerational factors in cognitive aging research.

